# Editors’ choice: the most valued articles published in the *European Journal of General Practice* in 2020

**DOI:** 10.1080/13814788.2021.1940516

**Published:** 2021-06-25

**Authors:** Carl Llor, Jelle Stoffers

**Affiliations:** on behalf of the Editorial Board of the European Journal of General Practice

Like previous years, the *European Journal of General Practice* (EJGP) editors evaluated the EJGP’s last year’s volume. In 2020, we published 36 articles, including 19 reports of original research, one systematic review, and four editorials. We had our share of COVID publications, including a background paper by WONCA Europe and several Letters to the Editor, following our first Editorial on this topic. We published three articles in the ongoing Series on eHealth. Authors came from various countries, including Belgium, Denmark, France, Greece, Hungary, Ireland, the Netherlands, Spain, and Turkey; there were a few international collaborative contributions [1].

The Editors each selected five articles they found most valuable and of which they established the scientific quality as ‘good’. We considered relevance for family medicine (general practice, primary care), originality, methodology and possible impact on practice or research. Overall, the highest-ranking paper was *‘Improving the quality of antibiotic prescribing through an educational intervention delivered through the out-of-hours general practice service in Ireland’* by *Nuala O’Connor et al.* In their paper, the authors describe how they improved the quality of antibiotics prescription during out-of-hours services in two Irish areas. They used a simple educational information tool targeted at GPs differentiating antibiotics into ‘red’ (to be avoided) and ‘green’ (recommended by national guidelines). At the end of each consultation, an electronic pop-up message was displayed in the patient management software if a decision to describe an antibiotic was made. The simple intervention reduced the proportion of prescribed ‘red’ antibiotics from 44% to 17% and increased the ‘green’ antibiotic prescriptions from 56% to 83%. We encourage the publication of sound studies that are innovative and relevant, like this one. This quality improvement study could easily be replicated in other settings, areas and countries, with a clear impact on public health. Congratulations to the whole team [2]!

In addition, we want to mention *Anne E. MacFarlane*’s opinion paper *‘Optimising individual and community involvement in health decision-making in general practice consultations and primary care settings: A way forward’* for her apparent attempt to discuss ‘three overlapping forms of patient involvement: shared decision-making in clinical care, community participation to develop services and Public and Patient Involvement in research’ [3].

Furthermore, we want to highlight the article *‘Lessons on the COVID-19 pandemic, for and by primary care professionals worldwide’* by *Salman Rawaf et al.*, which we consider the most valuable contribution on COVID-19 published in this Journal in 2020. The authors stress ‘the importance of maintaining access to primary care and ongoing management of all health problems that impact the local population’. They also provide an essential lesson on communication: ‘Users of information (public, patients, clinicians, or policymakers) all have their own information needs that cannot be served by the same measures’ [4].

You can read these and other published articles in the *European Journal of General Practice*.

Finally, we want to express our gratitude to all reviewers who have advised us during the year 2020. We thank you for your appreciated assistance! Your review reports were very valuable feedback to the authors and helped us to make our editorial decisions. Currently, the *European Journal of General Practice* accepts around 15% of all submitted manuscripts.

Although our database of authors and reviewers steadily increases, the Editors find it challenging to select referees within the Journal’s deadlines. Therefore, we sincerely hope that all reviewers want to continue their review work. If you would like to update your reviewer account, e.g. to specify your expertise, please visit http://mc.manuscript-central.com/ejgp to modify your profile. In addition, we invite all readers of the *European Journal of General Practice* interested in reviewing manuscripts for this Journal, to visit http://mc.manuscriptcentral.com/ejgp and to register as ‘new user’, i.e. to complete the details of their expertise.
